# Supplementary material for: The Systems Biology Research Tool: evolvable open-source software
Source: BMC Syst Biol. 2008 Jun 29;2:55. doi: 10.1186/1752-0509-2-55 (PMC2446383; doi:10.1186/1752-0509-2-55)
Supplement: Additional file 1 — SBRT Archive. An archive of the current version of the Systems Biology Research Tool. [file 1752-0509-2-55-S1.zip › sbrt-1.4.0/doc/developers_guide/api/sbrt/shell/mng/fba/FbaOptManager.html]

FbaOptManager


|  |  |  |
| --- | --- | --- |
| **Overview** \| **Package** \| **Class** \| **Use** \| **Tree** \| **Deprecated** \| **Index** \| **Help** | |  |
| **PREV CLASS**   **NEXT CLASS** | **FRAMES**    **NO FRAMES**     **All Classes** |  |
| SUMMARY: NESTED \| FIELD \| CONSTR \| METHOD | DETAIL: FIELD \| CONSTR \| METHOD |  |


---


## sbrt.shell.mng.fba Class FbaOptManager

```
java.lang.Object
  sbrt.shell.mng.AbstractProcessManager
      sbrt.shell.mng.fba.FbaManager
          sbrt.shell.mng.fba.FbaOptManager
```

**All Implemented Interfaces:**: KernelProcessManager<java.lang.String,java.lang.String>, ProcessManager<java.lang.String,java.lang.String>

---

``` public final class FbaOptManager extends FbaManager ```

This class is used to manage basic FBA optimization experiments.

**Author:**
:   This class was written and documented by Jeremiah Wright while in the
    Wagner lab.

---

| **Field Summary** | |
| --- | --- |
| `static java.lang.String` | `CONSTRAINTS_FILE`             The keyword used to denote a constraints file. |
| `static int` | `DEFAULT_SAFETY_LEVEL`             The default safety level used by `getSafetyLevel(ManagerInput, PrintWriter)`. |
| `static java.lang.String` | `OBJECTIVE_FUNCTION`             The keyword used to denote an objective function. |
| `static java.lang.String` | `OPTIMIZATION_SENSE`             The keyword used to denote the optimization sense, or direction. |
| `static UnmodifiableSet<java.lang.String>` | `optionalKeywords`             The set of keywords understood, but not required, by instances of this class. |
| `static UnmodifiableSet<java.lang.String>` | `requiredKeywords`             The set of keywords required to be present in the `ManagerInput` objects supplied to instances of this class. |
| `static java.lang.String` | `SAFETY_LEVEL`             The keyword used to denote the safety level. |

**Fields inherited from class sbrt.shell.mng.fba.FbaManager**

`CONSTRAINT_TOLERANCE, DEFAULT_CONSTRAINT_TOLERANCE, FLUX_VECTOR_FILE, FLUX_VECTOR_FILE_NAME, RXN_CATALYST_FILE, RXN_FILE, SYSTEM_SOLUTION_FILE`

**Fields inherited from class sbrt.shell.mng.AbstractProcessManager**

`ALGORITHM, COMPLETED, DATA_HEADERS, DEV_NULL, ELAPSED_TIME, FILE_FORMAT, INPUT_FILE, INPUT_FILE_FORMAT, INPUT_FILE_NAME_FILE, ITERATIONS, OUTPUT_FILE_FORMAT, OUTPUT_FILE_NAME, OUTPUT_FILE_NAME_FILE, PERCENT, PERCENT_COMPLETED, SEED, ZERO_CUTOFF`


**Constructor Summary**

`FbaOptManager()`


| **Method Summary** | |
| --- | --- |
| `void` | `execute(java.io.PrintWriter out)`             Executes the process this process manager controls, and writes the status or results of this process to the provided print writer. |
| `static FbaOptComputer` | `getComputer(ManagerInput<java.lang.String,java.lang.String> input, java.io.PrintWriter out)`             Creates and returns an FBA optimization computer based on the provided input. |
| `static FluxConstraints` | `getConstraints(ManagerInput<java.lang.String,java.lang.String> input, java.io.PrintWriter out, Fluxome fluxome)`             Opens the file denoted by `CONSTRAINTS_FILE` in the provided manager input and returns the set of flux constraints it contains. |
| `static FluxConstraints` | `getConstraints(java.lang.String fileName, Fluxome fluxome)`             Returns the set of constraints contained in the file with the provided name. |
| `static java.util.Set<java.lang.String>` | `getDataHeaders(ManagerInput<java.lang.String,java.lang.String> input, Fluxome fluxome)`             Parses and returns the value for `AbstractProcessManager.DATA_HEADERS` in the provided manager input. |
| `static java.util.LinkedHashSet<java.lang.String>` | `getDataHeaders(java.lang.String dataHeaders, Fluxome fluxome)`             Parses the provided data header string and returns the corresponding set. |
| `static IrrevRxnFluxome` | `getFluxome(ManagerInput<java.lang.String,java.lang.String> input, java.io.PrintWriter out)`             Creates and returns a fluxome based on the provided input. |
| `static IrrevRxnFluxome` | `getFluxome(ManagerInput<java.lang.String,java.lang.String> input, java.io.PrintWriter out, double tolerance)`             Creates and returns a fluxome based on the provided input. |
| `static MultiOutputFiles<ColumnOrFbaOptOutputFile,FbaOptComputer.Results>` | `getMultiColOrOutputFiles(ManagerInput<java.lang.String,java.lang.String> input, java.io.PrintWriter out, java.util.Iterator<java.lang.String> fileNameSource, java.lang.String fileNameSourceName, Fluxome fluxome)`             Please consult the source code for a complete description of this method's actions. |
| `static MultiOutputFiles<? extends FbaOptOutputFile,FbaOptComputer.Results>` | `getMultiFileOutput(ManagerInput<java.lang.String,java.lang.String> input, java.io.PrintWriter out, java.util.Iterator<java.lang.String> fileNameSource, java.lang.String fileNameSourceName, Fluxome fluxome)`             Please consult the source code for a complete description of this method's actions. |
| `static FbaOptOutputFile` | `getMultiResultsOutputFile(ManagerInput<java.lang.String,java.lang.String> input, java.io.PrintWriter out, Fluxome fluxome)`             Returns an empty output file that is suitable for storing multiple FBA optimization results. |
| `static FbaOptOutputFile` | `getMultiResultsOutputFile(java.lang.String fileName, FileFormat fileFormat, java.util.Set<java.lang.String> dataHeaders, Fluxome fluxome)`             Returns an empty output file that is suitable for storing multiple FBA optimization results. |
| `static MultiOutputFiles<RowOrFbaOptOutputFile,FbaOptComputer.Results>` | `getMultiRowOrOutputFiles(ManagerInput<java.lang.String,java.lang.String> input, java.io.PrintWriter out, java.util.Iterator<java.lang.String> fileNameSource, Fluxome fluxome)`             Please consult the source code for a complete description of this method's actions. |
| `static MultiOutputFiles<SerFbaOptOutputFile,FbaOptComputer.Results>` | `getMultiSerOutputFiles(ManagerInput<java.lang.String,java.lang.String> input, java.io.PrintWriter out, java.util.Iterator<java.lang.String> fileNameSource, java.lang.String fileNameSourceName, Fluxome fluxome)`             Please consult the source code for a complete description of this method's actions. |
| `static LinearComb<java.lang.String>` | `getObjectiveFunction(ManagerInput<java.lang.String,java.lang.String> input, java.io.PrintWriter out, Fluxome fluxome)`             Parses and returns the value for `OBJECTIVE_FUNCTION` in the provided manager input. |
| `static OptimizationSense` | `getOptimizationSense(ManagerInput<java.lang.String,java.lang.String> input)`             Parses and returns the value for `OPTIMIZATION_SENSE` in the provided manager input. |
| `UnmodifiableSet<java.lang.String>` | `getOptionalKeys()`             Returns the set of keywords the provided `ManagerInput` object does not necessarily have to contain. |
| `FbaOptimizationProcess` | `getProcess()`             Returns the process this process manager controls. |
| `UnmodifiableSet<java.lang.String>` | `getRequiredKeys()`             Returns the set of keywords the provided `ManagerInput` object must contain. |
| `static int` | `getSafetyLevel(ManagerInput<java.lang.String,java.lang.String> input, java.io.PrintWriter out)`             Parses and returns the value for `SAFETY_LEVEL` in the provided manager input. |
| `static FbaOptOutputFile` | `getSingleResultOutputFile(ManagerInput<java.lang.String,java.lang.String> input, java.io.PrintWriter out, Fluxome fluxome)`             Returns an empty output file that is suitable for storing a single FBA optimization result. |
| `static FbaOptOutputFile` | `getSingleResultOutputFile(java.lang.String fileName, FileFormat fileFormat, java.util.Set<java.lang.String> dataHeaders, Fluxome fluxome)`             Returns an empty output file that is suitable for storing a single FBA optimization result. |
| `static ApplicationException` | `interpret(ConstraintViolationException e, FbaProcess<?> experiment)`             Returns an `ApplicationException` whose message describes the error that ocurred. |
| `static ApplicationException` | `interpret(FbaOptException e, FbaProcess<?> experiment)`             Returns an `ApplicationException` whose message describes the error that ocurred. |
| `static ApplicationException` | `interpret(ProgramSolverException e)`             Returns an `ApplicationException` whose message describes the error that ocurred. |
| `static ApplicationException` | `interpret(java.lang.RuntimeException e, FbaProcess<?> experiment)`             Returns an `ApplicationException` whose message describes the error that ocurred. |
| `void` | `setInput(ManagerInput<java.lang.String,java.lang.String> input, java.io.PrintWriter out)`             Uses the provided input to prepare this process manager for execution. |

**Methods inherited from class sbrt.shell.mng.fba.FbaManager**

`getCatalysts, getCatalysts, getConstraintTolerance, getConstraintTolerance, getFluxome, getFluxome, getFluxome, getFluxome, getFluxomeSolution, getMultiFluxVectorOutputFile, getMultiFluxVectorOutputFile, getRxnNameExprFormat, getRxnNameHeaders, getRxnNameHeaders, getSingleFluxVectorOutputFile, getSingleFluxVectorOutputFile, printFluxomeInfo, writeFluxome, writeFluxome`

**Methods inherited from class sbrt.shell.mng.AbstractProcessManager**

`check, closeFile, closeRm, execute, getFileFormat, getFileFormat, getFileNameSource, getIterations, getSeed, getTextFileFormat, getTextFileFormat, getZeroCutoff, getZeroCutoff, parseInt, parseLong, printProgress, printProgress, printProgress, runProcess, setInput`

**Methods inherited from class java.lang.Object**

`clone, equals, finalize, getClass, hashCode, notify, notifyAll, toString, wait, wait, wait`

**Methods inherited from interface sbrt.shell.mng.ProcessManager**

`check, execute, setInput`

**Field Detail**

### DEFAULT\_SAFETY\_LEVEL

```
public static final int DEFAULT_SAFETY_LEVEL
```

:   The default safety level used by
    `getSafetyLevel(ManagerInput, PrintWriter)`.

    **See Also:**: Constant Field Values

---


### OBJECTIVE\_FUNCTION

```
public static final java.lang.String OBJECTIVE_FUNCTION
```

:   The keyword used to denote an objective function.

    **See Also:**: Constant Field Values

---


### CONSTRAINTS\_FILE

```
public static final java.lang.String CONSTRAINTS_FILE
```

:   The keyword used to denote a constraints file.

    **See Also:**: Constant Field Values

---


### OPTIMIZATION\_SENSE

```
public static final java.lang.String OPTIMIZATION_SENSE
```

:   The keyword used to denote the optimization sense, or direction.

    **See Also:**: Constant Field Values

---


### SAFETY\_LEVEL

```
public static final java.lang.String SAFETY_LEVEL
```

:   The keyword used to denote the safety level.

    **See Also:**: Constant Field Values

---


### requiredKeywords

```
public static final UnmodifiableSet<java.lang.String> requiredKeywords
```

:   The set of keywords required to be present in the `ManagerInput`
    objects supplied to instances of this class.

---


### optionalKeywords

```
public static final UnmodifiableSet<java.lang.String> optionalKeywords
```

:   The set of keywords understood, but not required, by instances of this
    class.


**Constructor Detail**

### FbaOptManager

```
public FbaOptManager()
```


**Method Detail**

### getObjectiveFunction

```
public static LinearComb<java.lang.String> getObjectiveFunction(ManagerInput<java.lang.String,java.lang.String> input,
                                                                java.io.PrintWriter out,
                                                                Fluxome fluxome)
```

:   Parses and returns the value for `OBJECTIVE_FUNCTION` in the
    provided manager input.

    :   **Parameters:**: `input` - the input containing a value for `OBJECTIVE_FUNCTION`.: `out` - the print writer to which a status message will be printed.: `fluxome` - the fluxome to which the objective function corresponds. **Returns:**: the parsed value for `OBJECTIVE_FUNCTION` in the provided input.

---


### getFluxome

```
public static IrrevRxnFluxome getFluxome(ManagerInput<java.lang.String,java.lang.String> input,
                                         java.io.PrintWriter out)
```

:   Creates and returns a fluxome based on the provided input. The values for
    `FbaManager.RXN_FILE`, `CONSTRAINTS_FILE`, and
    `FbaManager.CONSTRAINT_TOLERANCE` are parsed and combined to create
    the returned fluxome.

    :   **Parameters:**: `input` - the input containing values for the listed keywords.: `out` - the print writer to which status messages will be printed. **Returns:**: a fluxome based on the provided input. **See Also:**: `FbaManager.getFluxome(ManagerInput, PrintWriter)`, `FbaManager.getConstraintTolerance(ManagerInput, PrintWriter)`, `getConstraints(ManagerInput, PrintWriter, Fluxome)`, `Fluxome.replaceConstraints(FluxConstraints, double)`

---


### getFluxome

```
public static IrrevRxnFluxome getFluxome(ManagerInput<java.lang.String,java.lang.String> input,
                                         java.io.PrintWriter out,
                                         double tolerance)
```

:   Creates and returns a fluxome based on the provided input. The values for
    `FbaManager.RXN_FILE` and `CONSTRAINTS_FILE` are parsed and
    combined to create the returned fluxome.

    :   **Parameters:**: `input` - the input containing values for the listed keywords.: `out` - the print writer to which status messages will be printed.: `tolerance` - the constraint tolerance. **Returns:**: a fluxome based on the provided input. **See Also:**: `FbaManager.getFluxome(ManagerInput, PrintWriter)`, `getConstraints(ManagerInput, PrintWriter, Fluxome)`, `Fluxome.replaceConstraints(FluxConstraints, double)`

---


### getConstraints

```
public static FluxConstraints getConstraints(ManagerInput<java.lang.String,java.lang.String> input,
                                             java.io.PrintWriter out,
                                             Fluxome fluxome)
```

:   Opens the file denoted by `CONSTRAINTS_FILE` in the provided
    manager input and returns the set of flux constraints it contains.

    :   **Parameters:**: `input` - the input containing a value for `CONSTRAINTS_FILE`.: `out` - the print writer to which a status message will be printed.: `fluxome` - the fluxome to which the constraints correspond. **Returns:**: the set of flux constraints contained in the file indicated by `#CONSTRAINTS_FILE` in the provided input; or, if the input does not contain a value for `CONSTRAINTS_FILE`, an empty set of flux constraints. **See Also:**: `getConstraints(String, Fluxome)`

---


### getConstraints

```
public static FluxConstraints getConstraints(java.lang.String fileName,
                                             Fluxome fluxome)
```

:   Returns the set of constraints contained in the file with the provided
    name. This file must have the format of a row-oriented map file, and the
    lines must be parsable using `ConstraintsFileLineFormatV2`.

    :   **Parameters:**: `fileName` - the name of the file to be read.: `fluxome` - the fluxome to which the constraints correspond. **Returns:**: the set of constraints contained in the file with the provided name. **Throws:**: `java.lang.NullPointerException` - if any argument is `null`. **See Also:**: `RomiFile`

---


### getComputer

```
public static FbaOptComputer getComputer(ManagerInput<java.lang.String,java.lang.String> input,
                                         java.io.PrintWriter out)
```

:   Creates and returns an FBA optimization computer based on the provided
    input. The values for `FbaManager.RXN_FILE`, `CONSTRAINTS_FILE`,
    `FbaManager.CONSTRAINT_TOLERANCE`, `SAFETY_LEVEL`,
    `ProgramSolverFactory.SOLVER`, and
    `ProgramSolverFactory.PARAM_FILE` are parsed and combined to create
    the returned optimization computer.

    :   **Parameters:**: `input` - the input containing values for the listed keywords.: `out` - the print writer to which status messages will be printed. **Returns:**: an FBA optimization computer based on the provided input. **See Also:**: `getFluxome(ManagerInput, PrintWriter)`, `getSafetyLevel(ManagerInput, PrintWriter)`, `FbaManager.getConstraintTolerance(ManagerInput, PrintWriter)`, `ProgramSolverFactory.make(ManagerInput, PrintWriter)`

---


### getSafetyLevel

```
public static int getSafetyLevel(ManagerInput<java.lang.String,java.lang.String> input,
                                 java.io.PrintWriter out)
```

:   Parses and returns the value for `SAFETY_LEVEL` in the provided
    manager input.

    :   **Parameters:**: `input` - the input containing a value for `SAFETY_LEVEL`.: `out` - the print writer to which a status message will be printed. **Returns:**: the parsed value for `SAFETY_LEVEL` in the provided input, or `DEFAULT_SAFETY_LEVEL` if the provided input does not contain such a value.

---


### getOptimizationSense

```
public static OptimizationSense getOptimizationSense(ManagerInput<java.lang.String,java.lang.String> input)
```

:   Parses and returns the value for `OPTIMIZATION_SENSE` in the
    provided manager input.

    :   **Parameters:**: `input` - the input containing a value for `OPTIMIZATION_SENSE`. **Returns:**: the parsed value for `OPTIMIZATION_SENSE` in the provided manager input.

---


### interpret

```
public static ApplicationException interpret(java.lang.RuntimeException e,
                                             FbaProcess<?> experiment)
```

:   Returns an `ApplicationException` whose message describes the error
    that ocurred.

    :   **Parameters:**: `e` - a runtime exception thrown during an FBA optimization.: `experiment` - the FBA experiment from which the exception was thrown. **Returns:**: an `ApplicationException` wrapping the provided exception. **Throws:**: `java.lang.RuntimeException` - the original exception if it cannot be intrepretted.

---


### interpret

```
public static ApplicationException interpret(ProgramSolverException e)
```

:   Returns an `ApplicationException` whose message describes the error
    that ocurred.

    :   **Parameters:**: `e` - a program solver exception. **Returns:**: an `ApplicationException` wrapping the provided exception.

---


### interpret

```
public static ApplicationException interpret(ConstraintViolationException e,
                                             FbaProcess<?> experiment)
```

:   Returns an `ApplicationException` whose message describes the error
    that ocurred.

    :   **Parameters:**: `e` - a constraint violation exception.: `experiment` - the FBA experiment from which the exception was thrown. **Returns:**: an `ApplicationException` wrapping the provided exception.

---


### interpret

```
public static ApplicationException interpret(FbaOptException e,
                                             FbaProcess<?> experiment)
```

:   Returns an `ApplicationException` whose message describes the error
    that ocurred.

    :   **Parameters:**: `e` - an FBA optimization exception.: `experiment` - the FBA experiment from which the exception was thrown. **Returns:**: an `ApplicationException` wrapping the provided exception.

---


### getDataHeaders

```
public static java.util.Set<java.lang.String> getDataHeaders(ManagerInput<java.lang.String,java.lang.String> input,
                                                             Fluxome fluxome)
```

:   Parses and returns the value for `AbstractProcessManager.DATA_HEADERS` in the provided
    manager input. This method uses `FbaOptHeaderParserV1` to perform
    the parsing.

    :   **Parameters:**: `input` - the input containing a value for `DATA_HEADERS`.: `fluxome` - the fluxome to which the data headers correspond. **Returns:**: the parsed value for `AbstractProcessManager.DATA_HEADERS` in the provided manager input; or the set of all possible data headers if the input contains no such value.

---


### getDataHeaders

```
public static java.util.LinkedHashSet<java.lang.String> getDataHeaders(java.lang.String dataHeaders,
                                                                       Fluxome fluxome)
```

:   Parses the provided data header string and returns the corresponding set.
    This method uses `FbaOptHeaderParserV1` to perform the parsing.

    :   **Parameters:**: `dataHeaders` - a string containing data headers.: `fluxome` - the fluxome to which the data headers correspond. **Returns:**: the set of data headers parsed from the provided string; or the set of all possible data headers if the provided string is `null`.

---


### getMultiResultsOutputFile

```
public static FbaOptOutputFile getMultiResultsOutputFile(ManagerInput<java.lang.String,java.lang.String> input,
                                                         java.io.PrintWriter out,
                                                         Fluxome fluxome)
```

:   Returns an empty output file that is suitable for storing multiple FBA
    optimization results. The values for `AbstractProcessManager.OUTPUT_FILE_NAME`,
    `AbstractProcessManager.DATA_HEADERS`, and
    `AbstractProcessManager.FILE_FORMAT` in the provided input are
    parsed and used to call the method
    `getMultiResultsOutputFile(String, FileFormat, Set, Fluxome)`.

    :   **Parameters:**: `input` - the input containing values for the listed keywords.: `out` - the print writer to which status messages will be printed.: `fluxome` - the fluxome to which the results correspond. **Returns:**: an empty output file that is suitable for storing multiple FBA optimization results. **See Also:**: `getDataHeaders(ManagerInput, Fluxome)`, `AbstractProcessManager.getFileFormat(ManagerInput)`

---


### getMultiResultsOutputFile

```
public static FbaOptOutputFile getMultiResultsOutputFile(java.lang.String fileName,
                                                         FileFormat fileFormat,
                                                         java.util.Set<java.lang.String> dataHeaders,
                                                         Fluxome fluxome)
```

:   Returns an empty output file that is suitable for storing multiple FBA
    optimization results. This method uses `FbaOptFileLineFormatV1`.

    :   **Parameters:**: `fileName` - the name of the file to be created.: `fileFormat` - the format of the file to be created.: `dataHeaders` - the set of data headers to be written to the file.: `fluxome` - the fluxome to which the results correspond. **Returns:**: an empty output file that is suitable for storing multiple FBA optimization results.

---


### getSingleResultOutputFile

```
public static FbaOptOutputFile getSingleResultOutputFile(ManagerInput<java.lang.String,java.lang.String> input,
                                                         java.io.PrintWriter out,
                                                         Fluxome fluxome)
```

:   Returns an empty output file that is suitable for storing a single FBA
    optimization result. The values for `AbstractProcessManager.OUTPUT_FILE_NAME`,
    `AbstractProcessManager.DATA_HEADERS`, and
    `AbstractProcessManager.FILE_FORMAT` in the provided input are
    parsed and used to call the method
    `getSingleResultOutputFile(String, FileFormat, Set, Fluxome)`.

    :   **Parameters:**: `input` - the input containing values for the listed keywords.: `out` - the print writer to which status messages will be printed.: `fluxome` - the fluxome to which the results correspond. **Returns:**: an empty output file that is suitable for storing a single FBA optimization result. **See Also:**: `getDataHeaders(ManagerInput, Fluxome)`, `AbstractProcessManager.getFileFormat(ManagerInput)`

---


### getSingleResultOutputFile

```
public static FbaOptOutputFile getSingleResultOutputFile(java.lang.String fileName,
                                                         FileFormat fileFormat,
                                                         java.util.Set<java.lang.String> dataHeaders,
                                                         Fluxome fluxome)
```

:   Returns an empty output file that is suitable for storing a single FBA
    optimization result. This method uses `FbaOptFileLineFormatV1`.

    :   **Parameters:**: `fileName` - the name of the file to be created.: `fileFormat` - the format of the file to be created.: `dataHeaders` - the set of data headers to be written to the file.: `fluxome` - the fluxome to which the results correspond. **Returns:**: an empty output file that is suitable for storing a single FBA optimization result.

---


### getMultiFileOutput

```
public static MultiOutputFiles<? extends FbaOptOutputFile,FbaOptComputer.Results> getMultiFileOutput(ManagerInput<java.lang.String,java.lang.String> input,
                                                                                                     java.io.PrintWriter out,
                                                                                                     java.util.Iterator<java.lang.String> fileNameSource,
                                                                                                     java.lang.String fileNameSourceName,
                                                                                                     Fluxome fluxome)
```

:   Please consult the source code for a complete description of this
    method's actions.

    :   **Parameters:**: `input` -: `out` -: `fileNameSource` -: `fileNameSourceName` -: `fluxome` - **Returns:**: an object used to write multiple output files.

---


### getMultiSerOutputFiles

```
public static MultiOutputFiles<SerFbaOptOutputFile,FbaOptComputer.Results> getMultiSerOutputFiles(ManagerInput<java.lang.String,java.lang.String> input,
                                                                                                  java.io.PrintWriter out,
                                                                                                  java.util.Iterator<java.lang.String> fileNameSource,
                                                                                                  java.lang.String fileNameSourceName,
                                                                                                  Fluxome fluxome)
```

:   Please consult the source code for a complete description of this
    method's actions.

    :   **Parameters:**: `input` -: `out` -: `fileNameSource` -: `fileNameSourceName` -: `fluxome` - **Returns:**: an object used to write multiple output files.

---


### getMultiColOrOutputFiles

```
public static MultiOutputFiles<ColumnOrFbaOptOutputFile,FbaOptComputer.Results> getMultiColOrOutputFiles(ManagerInput<java.lang.String,java.lang.String> input,
                                                                                                         java.io.PrintWriter out,
                                                                                                         java.util.Iterator<java.lang.String> fileNameSource,
                                                                                                         java.lang.String fileNameSourceName,
                                                                                                         Fluxome fluxome)
```

:   Please consult the source code for a complete description of this
    method's actions.

    :   **Parameters:**: `input` -: `out` -: `fileNameSource` -: `fileNameSourceName` -: `fluxome` - **Returns:**: an object used to write multiple output files.

---


### getMultiRowOrOutputFiles

```
public static MultiOutputFiles<RowOrFbaOptOutputFile,FbaOptComputer.Results> getMultiRowOrOutputFiles(ManagerInput<java.lang.String,java.lang.String> input,
                                                                                                      java.io.PrintWriter out,
                                                                                                      java.util.Iterator<java.lang.String> fileNameSource,
                                                                                                      Fluxome fluxome)
```

:   Please consult the source code for a complete description of this
    method's actions.

    :   **Parameters:**: `input` -: `out` -: `fileNameSource` -: `fluxome` - **Returns:**: an object used to write multiple output files.

---


### getRequiredKeys

```
public UnmodifiableSet<java.lang.String> getRequiredKeys()
```

:   Returns the set of keywords the provided `ManagerInput` object must
    contain.

    :   **Returns:**: the set of keywords the provided `ManagerInput` object must contain.

---


### getOptionalKeys

```
public UnmodifiableSet<java.lang.String> getOptionalKeys()
```

:   Returns the set of keywords the provided `ManagerInput` object does
    not necessarily have to contain.

    :   **Returns:**: the set of keywords the provided `ManagerInput` object does not necessarily have to contain.

---


### getProcess

```
public FbaOptimizationProcess getProcess()
```

:   Returns the process this process manager controls.

    :   **Returns:**: the process this process manager controls. **Throws:**: `java.lang.IllegalStateException` - if no input was provided to this process manager via `AbstractProcessManager.setInput(ManagerInput)` or `setInput(ManagerInput, PrintWriter)`

---


### setInput

```
public void setInput(ManagerInput<java.lang.String,java.lang.String> input,
                     java.io.PrintWriter out)
```

:   Uses the provided input to prepare this process manager for execution. As
    the input is processed, informative messages are printed to the provided
    print writer.

    :   **Parameters:**: `input` - all of the information required to prepare this process manager for execution.: `out` - the print writer to which all messages will be printed.

---


### execute

```
public void execute(java.io.PrintWriter out)
```

:   Executes the process this process manager controls, and writes the status
    or results of this process to the provided print writer.

    :   **Parameters:**: `out` - the print writer where status messages will be printed. **Throws:**: `java.lang.IllegalStateException` - if no input was provided to this process manager via `AbstractProcessManager.setInput(ManagerInput)` or `setInput(ManagerInput, PrintWriter)`


---


|  |  |  |
| --- | --- | --- |
| **Overview** \| **Package** \| **Class** \| **Use** \| **Tree** \| **Deprecated** \| **Index** \| **Help** | |  |
| **PREV CLASS**   **NEXT CLASS** | **FRAMES**    **NO FRAMES**     **All Classes** |  |
| SUMMARY: NESTED \| FIELD \| CONSTR \| METHOD | DETAIL: FIELD \| CONSTR \| METHOD |  |


---
